# Suture Augmented Ulnar Collateral Ligament Reconstruction: A Detailed Technique

**DOI:** 10.1002/atn2.70175

**Published:** 2026-07-14

**Authors:** Carson H. Gardner, Seena Sebt, Kenneth H. Akizuki

**Affiliations:** ^1^ San Francisco Orthopaedic Residency Program – UCSF Health St. Mary's San Francisco California U.S.A.; ^2^ Cedars Sinai Medical Center Los Angeles California U.S.A.; ^3^ West Coast Sports Institute San Francisco California U.S.A.

## Abstract

Suture augmentation is frequently being used for medial ulnar collateral ligament (UCL) repair and many surgeons now augment UCL reconstructions with a high strength suture. This article and video describe the technique of suture augmented UCL reconstruction. The technique uses an autograft from palmaris or hamstring that is supported by a suture across the reconstruction. The graft is further modified using multiple points of suture augmentation to strengthen the reconstruction including a 1.6 mm FiberTak at the humerus to bolster the proximal aspect of the reconstruction. The graft is also incorporated into a side to side repair of the native ligament. The graft and suture are both tensioned over the medial epicondyle using a modified docking technique. Suture augmented reconstruction of the UCL is growing in popularity, yet the current literature is lacking in studies investigating clinical outcomes.

## Graphical abstract

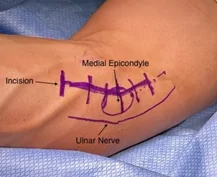

**VIDEO 1:** This video shows a suture augmented hybrid ulnar collateral ligament reconstruction performed on a right arm. A palmaris graft is harvested and used to reconstruct the ulnar collateral ligament. The arm is viewed from the axillary position on a hand table. Video content can be viewed at https://doi.org/10.1002/atn2.70175.

Ulnar collateral ligament (UCL) injuries are increasingly prevalent among throwing athletes, particularly baseball pitchers, due to the repetitive valgus stress placed on the elbow during overhead throwing. Depending on the athlete and severity of the injury, some UCL tears can be managed conservatively with rest, bracing, and physical therapy.^1^ Nonoperative management may also include platelet‐rich plasma (PRP) injections, though success rates vary.^2^, ^3^, ^4^ However, in elite overhead athletes or when conservative measures fail, surgical intervention is required in the form of UCL reconstruction or repair, each with distinct advantages and limitations.

UCL repair techniques are gaining traction for select injuries, particularly in younger athletes with higher quality ligament tissue.^5^, ^6^, ^7^ Studies have shown that augmented repair shows superior biomechanical strength compared with isolated repair.^8^, ^9^, ^10^, ^11^ However, repair may not be a good option for patients with poor quality ligament tissue and some research has shown higher rates of revision with repair.^12^ Given the success of UCL repairs augmented with high strength suture, some surgeons have begun augmenting their UCL reconstructions with suture as well.^13^, ^14^, ^15^ Given the recent adoption of this strategy, a handful of surgeons have reported their techniques in the literature.^13^, ^14^, ^15^ The purpose of this article is to describe the technique of a busy UCL surgeon performing hybrid UCL reconstruction.

## SURGICAL TECHNIQUE

### Patient Positioning

The patient is positioned supine on a standard operating table with a hand table. A sterile tourniquet is applied to the upper arm. The medial epicondyle is marked as well as the course of the ulnar nerve (Figure 1). The ulnar nerve is palpated and evaluated to ensure it does not subluxate. A curvilinear incision is marked out making an approximately 30° curve over the medial epicondyle.

**FIGURE 1:**
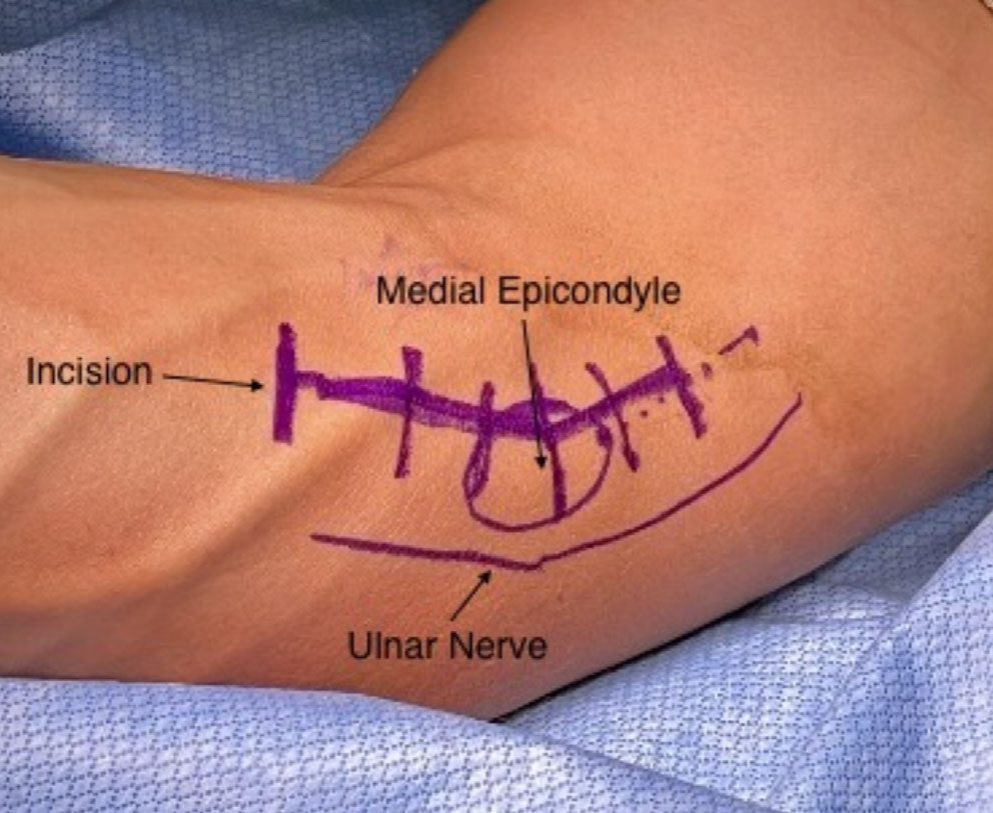
Viewing the medial aspect of a right elbow with distal to the left and proximal to the right. The medial epicondyle and ulnar nerve are marked and a curvilinear incision is marked anterior to the medial epicondyle.

### Approach

The arm is exsanguinated, and the tourniquet is inflated. An incision is made through skin, and dissection is carried down through the subcutaneous tissue taking care to preserve any subcutaneous veins and branches of the medial antebrachial cutaneous nerve. Any large branches of the medial antebrachial cutaneous nerve crossing the exposure are retracted with a vessel loop (Video 1). The flexor fascia is exposed and incised between the 2 heads of the flexor carpi ulnaris. The flexor carpi ulnaris is then dissected bluntly and with bipolar cautery to expose the sublime tubercle distally (Figure 2). Dissection is carried along the UCL proximally to the medial epicondyle. The ulnar nerve is then exposed and protected. The UCL is incised longitudinally, exposing the underlying ulnohumeral joint. At this point the quality of the ligament can be best evaluated (Figure 3).

**FIGURE 2:**
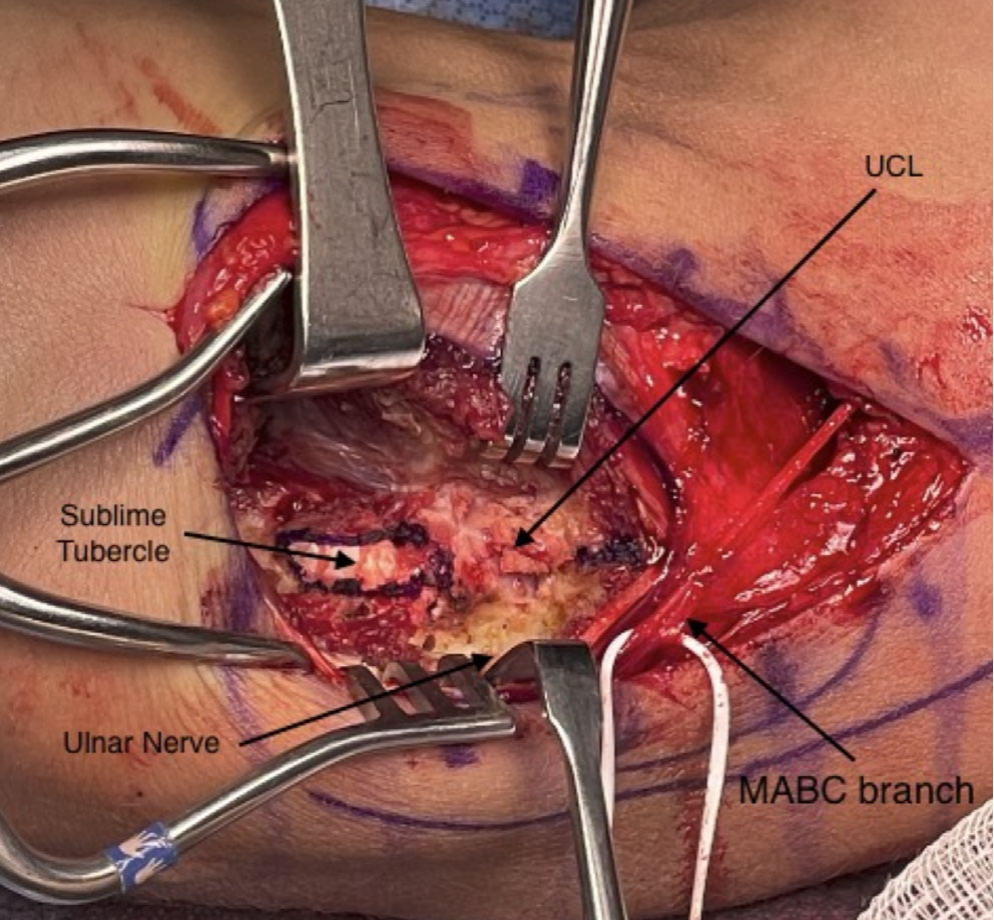
Viewing the medial aspect of a right elbow with distal to the left and proximal to the right. The sublime tubercle is visualized in the distal part of the incision with the native ulnar collateral ligament (UCL) extending proximally from it. The ulnar nerve is always identified in order to safely place the medial tunnel at the sublime tubercle. Branches of the medial antebrachial cutaneous nerve (MABC) are retracted with a vessel loop.

**FIGURE 3:**
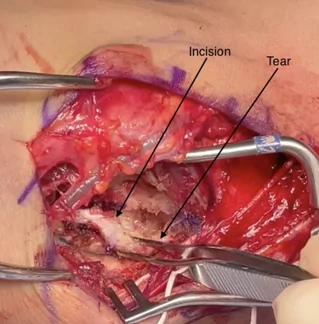
Viewing the medial aspect of a right elbow with distal to the left and proximal to the right. A longitudinal incision is made in the ulnar collateral ligament (incision). At the proximal aspect of the ligament a transverse tear can be seen (tear).

A 3.5 mm SwiveLock anchor (Arthrex, Naples, FL) loaded with 2 mm collagen coated FiberTape (Arthrex, Naples, FL) and 0 FiberWire (Arthrex, Naples, FL) is placed at the sublime tubercle. This anchor should be placed parallel to the joint surface and as near to it as possible to preserve space for the 2 distal tunnels. Next, two 3.5 mm converging tunnels are drilled just distal to the previously placed anchor in the sublime tubercle, one anteriorly and 1 posteriorly (Figure 4). It is critical to expose the ulnar nerve distally at the sublime tubercle, as the posterior tunnel is immediately adjacent to the nerve. A 4.5 mm unicortical tunnel is drilled in the medial epicondyle. Next, 3.5 mm (medial) and 2.5 mm (anterior) tunnels are drilled proximally to distally to converge with the 4.5 mm tunnel (Video 1). If the ulnar nerve is transposed, both tunnels can be shifted slightly posteriorly to avoid irritation of the nerve on suture knots. There should be a bridge of at least 15 mm between the openings of the 2 proximal tunnels. A 1.6 mm FiberTak (Arthrex, Naples, FL) anchor is placed just proximal and medial to the distal tunnel in the humerus to facilitate later repair of the proximal aspect of ligament.

**FIGURE 4:**
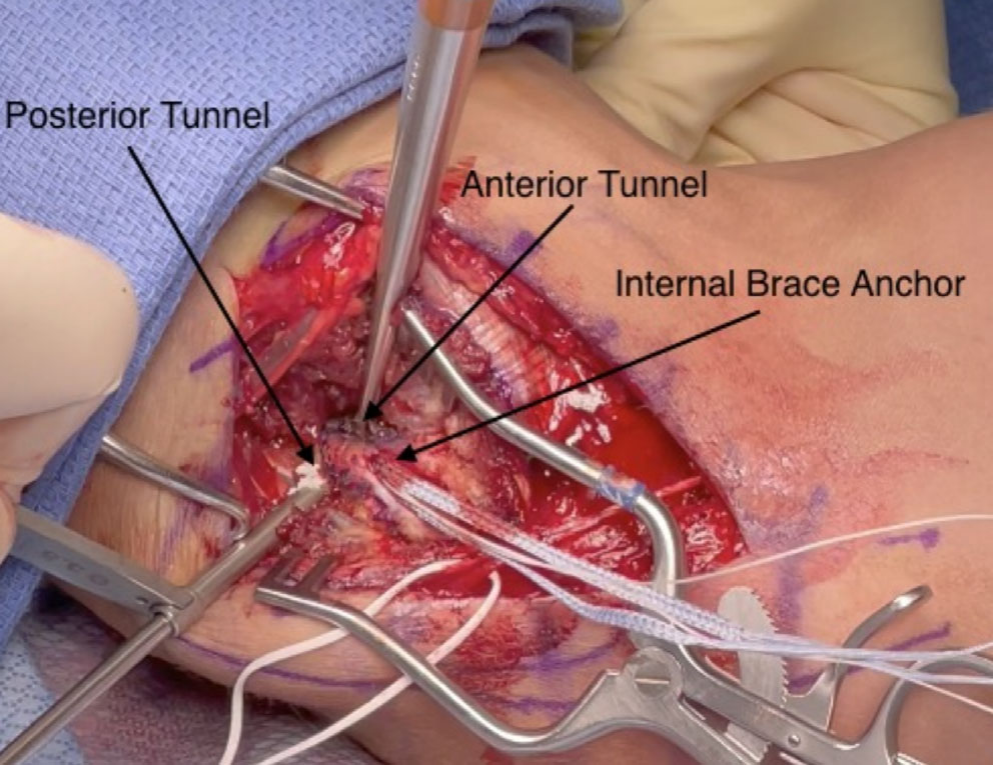
Viewing the medial aspect of a right elbow with distal to the left and proximal to the right. The SwiveLock anchor for the FiberTape is placed at the sublime tubercle adjacent to the joint. Immediately distal to the FiberTape anchor are anterior and medial converging tunnels for the graft.

### Palmaris Harvest

The palmaris tendon is harvested using a three‐incision technique. If the ipsilateral palmaris is not available, the contralateral palmaris can be harvested, or hamstring tendon if neither palmaris is available. Three small transverse incisions are planned along the tendon starting at the wrist crease and proceeding proximally in 7 cm intervals. The distal incision is made first and dissection is carried down to the tendon. Great caution must be employed to ensure that the tendon is correctly identified, as the median nerve runs deep to the tendon in this location. A tonsil is passed under the tendon to hold it out of the skin on tension, and the tendon is then identified in the next 2 proximal incisions (Figure 5). The tendon is released distally, pulled through to the most proximal incision, and a 2‐0 FiberLoop (Arthrex, Naples, FL) is whipstitched through the distal end of the tendon. The proximal end of the tendon is then cut.

**FIGURE 5:**
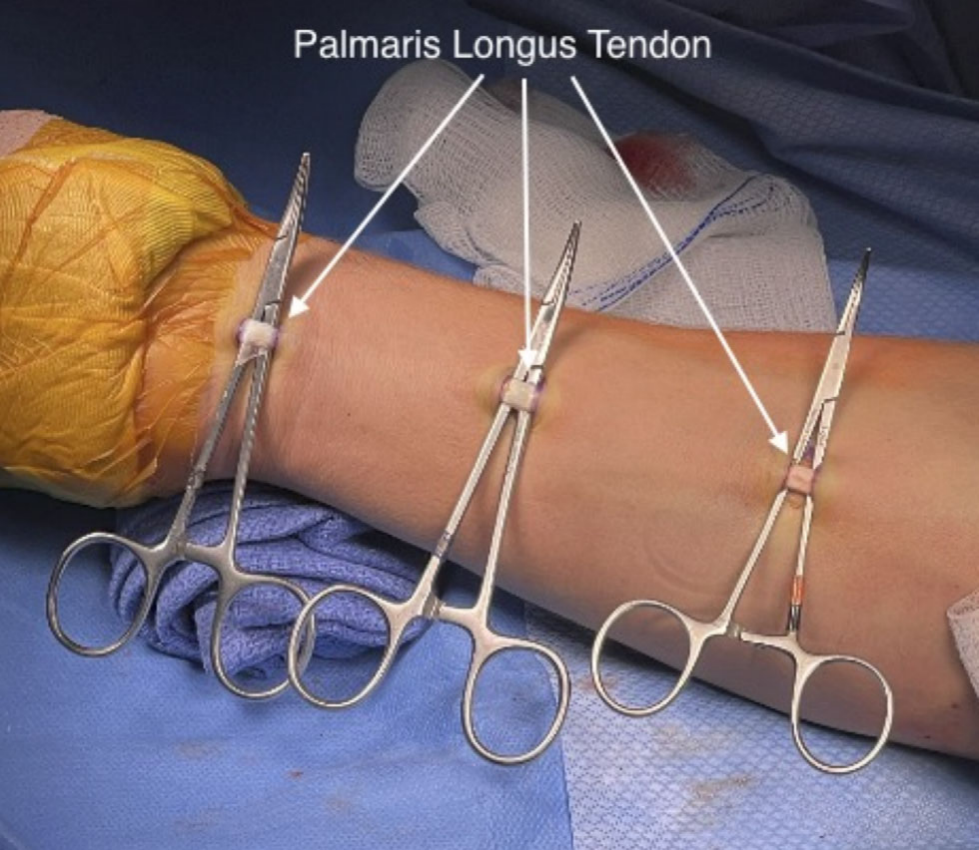
Viewing the medial aspect of a right elbow with distal to the left and proximal to the right. The palmaris longus is harvested using a three‐incision technique spaced 7 cm apart. Correct identification of the tendon is critical as the median nerve is deep to the tendon.

The tendon is lubricated with mineral oil and passed through the distal tunnel at the sublime tubercle. At this point, the native UCL tendon is repaired side to side with a running Krackow 1.3 mm SutureTape (Arthrex, Naples, FL) over the collagen coated FiberTape, imbricating the FiberTape into the tendon repair (Video 1). The 0 FiberWire from the distal SwiveLock anchor is then used to repair the distal ligament side to side. Next, the whipstitched tail of the graft, and the anterior tails of the FiberTape and SutureTape are passed through the medial tunnel. The other end of the graft is then marked and cut at a point so that a small section of it will enter the distal tunnel. This end is also whipstitched and then passed through the anterior tunnel at the medial epicondyle along with the posterior limbs of the FiberTape and SutureTape. The elbow is ranged with the graft on tension in order to test isometry and cycle the graft. The FiberTape, graft, and SutureTape are then tensioned and tied over the medial epicondyle in that order with the elbow in 50° of flexion and supination with a varus stress. The sutures from the 1.6 mm Fibertak are used to repair the proximal aspect of the graft together side to side. Additional side to side repair is performed with 0 vicryl (Johnson & Johnson, New Brunswick, NJ) (Figure 6).

**FIGURE 6:**
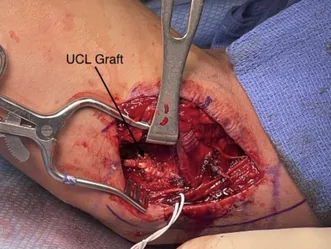
Viewing the medial aspect of a right elbow with distal to the left and proximal to the right. The implanted ulnar collateral ligament (UCL) graft is augmented with side‐to‐side sutures.

The flexor fascia is then repaired with running 0 vicryl in a locked fashion. The skin is repaired in layers followed by skin glue and sterile strips. A posterior long arm splint is applied in 80° of flexion and neutral forearm rotation. The advantages and disadvantages of our technique are summarized in Table 1, and the pearls and pitfalls are detailed in Table 2.

**TABLE 1:** Advantages and Disadvantages of Hybrid Reconstruction

**Advantages**	**Disadvantages**
• Improved outcomes over repair in mid‐substance tears with poor ligament quality	• Need for obtaining autograft
• Reconstruction is protected by the FiberTape as it heals	• Increased operative time
• Tension can be adjusted with both the graft and FiberTrape as they're tied over the medial epicondyle	• Longer recovery compared with repair
• Graft is augmented in numerous locations to aid in healing and prevent re‐tear	• Requires numerous tunnels at the sublime tubercle resulting in difficult tunnel placement
	• Risk of fracture at medial epicondyle

**TABLE 2:** Pearls and Pitfalls of Hybrid Reconstruction

**Pearls**	**Pitfalls**
• A sterile tourniquet maximizes the surgical field	• If the ulnar nerve is transposed, then the proximal medial epicondyle tunnels should both be translated posteriorly to avoid the nerve resting on the knot stack
• Positioning of the arm right at the axillary edge of the hand table facilitates the surgeon dropping their hand to drill the more medial tunnel at the sublime tubercle	
• Bending the Hewson suture passer allows for easier passage of sutures through the tunnels at the sublime tubercle	
• Placement of curettes in the tunnels aids in trajectory as the converging tunnels are drilled	
• Mineral oil is useful to pass the graft smoothly through tunnels	
• The graft and FiberTape are both tensioned in 50° of flexion, supination, and a varus force at the elbow	

### Rehabilitation

The arm is immobilized in a splint for 1 week after which the patient is advanced to a hinged elbow brace. Patients are limited in flexion to 90° for the first 3 weeks then may advance range of motion as tolerated. An interval throwing program is started at approximately 5 months and return to play typically occurs around 12 months after surgery.

## DISCUSSION

Management of UCL injuries has advanced since the first reconstruction was performed by Dr. Jobe.^16^ Recently, UCL repair has regained popularity with the advent of suture augmentation.^5^, ^6^, ^7^ Given the success of suture augmented repair for certain UCL injuries, many high volume UCL surgeons are now augmenting their reconstructions with high strength suture.^13^, ^15^, ^17^, ^18^ This technical note describes the UCL reconstruction technique of the senior author in detail. The reconstruction is augmented with a FiberTape anchored at the sublime tubercle and included in the modified docking technique at the humerus. The reconstruction is also augmented with a side‐to‐side repair of the proximal graft using a FiberTak suture, the distal graft with an 0 FiberWire anchored in the distal SwiveLock anchor, and along the graft with free 0 vicryl sutures. Although suture augmented UCL reconstruction is popular, the literature is lacking in clinical studies investigating outcomes, and future studies are warranted.

## DISCLOSURES

The authors (C.H.G., S.S., K.H.A.) declare that they have no known competing financial interests or personal relationships that could have appeared to influence the work reported in this article.
